# Quality of the Indoor Environment in Elderly Care Centers in Two Cities in Central Portugal: Viseu and Covilhã

**DOI:** 10.3390/ijerph16203801

**Published:** 2019-10-09

**Authors:** Manuel Pinto, João Lanzinha, João Viegas, Catarina Infante, Tiago Freire

**Affiliations:** 1 School of Technology & Management (ESTGV)—CONSTRUCT-LFC, Campus de Repeses, 3504-510 Viseu, Portugal; 2 LABSED-UBIMedical, C-made—Centre of Materials and Building Technologies, Faculty of Engineering, University of Beira Interior (UBI), Rua Marquês D’Ávila e Bolama, 6201-001 Covilhã, Portugal; joao.lanzinha@ubi.pt; 3 National Laboratory for Civil Engineering (LNEC), Av. do Brasil, 101, 1700-066 Lisboa, Portugal; jviegas@lnec.pt; 4 Master in Construction Engineering and Rehabilitation by the School of Technology & Management (ESTGV), Campus de Repeses, 3504-510 Viseu, Portugal; katarina.infante@gmail.com; 5 Master in Civil Engineering by University of Beira Interior (UBI), Rua Marquês D’Ávila e Bolama, 6201-001 Covilhã, Portugal; tiago.sf6@gmail.com

**Keywords:** Indoor Environment Quality (IEQ), Indoor Air Quality (IAQ), Elderly Care Centers (ECC)

## Abstract

Assessments of Indoor Environment Quality (IEQ) present a very significant challenge when analyses are undertaken mainly in buildings that include a particularly sensitive and vulnerable population, such as elderly people. In order to maintain an indoor environment that is adequate for occupants, it is necessary to comply with a set of requirements (for TVOC, the Portuguese threshold values) regarding concentrations of airborne pollutants and hygrothermal comfort conditions. This paper studies IEQ in compartments in 3 buildings in two cities in central Portugal, Viseu and Covilhã, which hold elderly care centers. The following environmental parameters were continuously recorded: air temperature, relative humidity, concentration of carbon dioxide, formaldehyde, and total volatile organic compounds and ventilation rates. An analysis of the obtained results was performed, taking recommended guidelines and threshold values into account, thus making it possible to evaluate the IEQ conditions and hygrothermal comfort in the selected indoor spaces. On the basis of the conclusions reached and the observed problems of hygrothermal comfort and indoor pollutants in the indoor spaces, a number of recommendations are proposed, specifically in terms of climate control, ventilation, and maintenance, in order to obtain an overall improvement of IEQ.

## 1. Introduction

In developed countries, people spend more than 90% of their time indoors. Indoor environmental conditions are strongly related to health, well-being, and overall performance [1]. With people spending so much time inside buildings, the issue of Indoor Environment Quality (IEQ) becomes especially important; therefore, a set of functional requirements exist to provide increased occupant comfort. In addition to the need to satisfy occupants’ comfort requirements, special attention must be paid to their health, since inadequate IEQ can negatively influence the occupants’ quality of life, affecting their health status.

Although IEQ plays an important role in all types of buildings, particular consideration should be paid to Elderly Care Centers (ECC). The perception of the elderly as a risk group with regard to inadequate indoor environments, due mainly to the presence of pollutants in the indoor air, is essentially due to the fact that the elderly population’s immune defenses are reduced, and many of them suffer from multiple chronic diseases [2]. Every building must therefore be designed, constructed, and maintained in order to provide adequate conditions of comfort [3].

With regard to the age pyramid in Portugal, approximately 19% of the population is aged 65 or over. The Aging Index, i.e., the relationship between the number of people over 65 years of age and the number under the age of 14, has shown an increasing trend in recent decades, mainly due to the decrease in the birth rate [4]. ECC have the potential to influence residents’ lives socially, physically, and psychologically. Older people may be particularly at risk from the effects of air pollutants, even at low concentrations, because of their reduced immune defenses, as well as any underlying chronic diseases [5]. In addition, the results of some studies on thermal sensation indicate that older people prefer higher temperatures, in contrast with young adults [6,7,8]. In short, older people tend to have different thermal sensations and preferences compared to younger people. This, in turn, affects the way older people respond to changes in the thermal conditions around them [9,10].

Studies in ECC are rare, perhaps because the premise that in these places, problems associated with the Indoor Air Quality (IAQ) are less important, due to the relatively low occupancy density. The most common studies in this field are related to comfort analyses [11]. However, in the last few years, some research on IAQ in premises for elderly people has been carried out. Walgraeve et al. [12] improved the sampling technique for VOC and applied their new technique in an assessment of IAQ in Flemish homes for the elderly. Almeida-Silva, Wolterbeek and Almeida [13] characterized the indoor air quality in ECC in order to assess residents’ daily exposure to air pollutants, and to identify microenvironments with highest levels of impact on the elderly. Shao et al. [14] referred to the importance of air filtration as a means to mitigate reduced indoor air pollution levels.

More research is needed to better describe the IAQ where the elderly people are passing most of their time. This research is intended to contribute to that effort by providing information on elderly care premises in Portugal.

In order to maintain an indoor environment with acceptable air quality and levels of thermal comfort, it is necessary to comply with the reference levels for temperature and relative humidity (*T*_int_ e *RH*), with minimum values of air change rates (ACH) and with maximum values of concentrations of indoor air pollutants. Portuguese regulations provide parameters for thermal considerations in buildings and IEQ, with the exception of RH, for which European standardization provides recommendations.

Compliance with the reference levels and guideline and threshold values still requires a careful analysis of indoor air ventilation conditions. Indoor air ventilation plays a very important role, not only for IEQ, since the levels of pollutants are controlled through ACH, but also in the conservation of the building, often preventing the development of pathologies related to humidity; these pathologies may cause the degradation of certain components of buildings which, in turn, leads to a decrease in IEQ for occupants. In buildings, the use of ventilation must also be adequate, so that thermal discomfort is avoided, but also to avoid excessive energy consumption.

**Figure 1:**
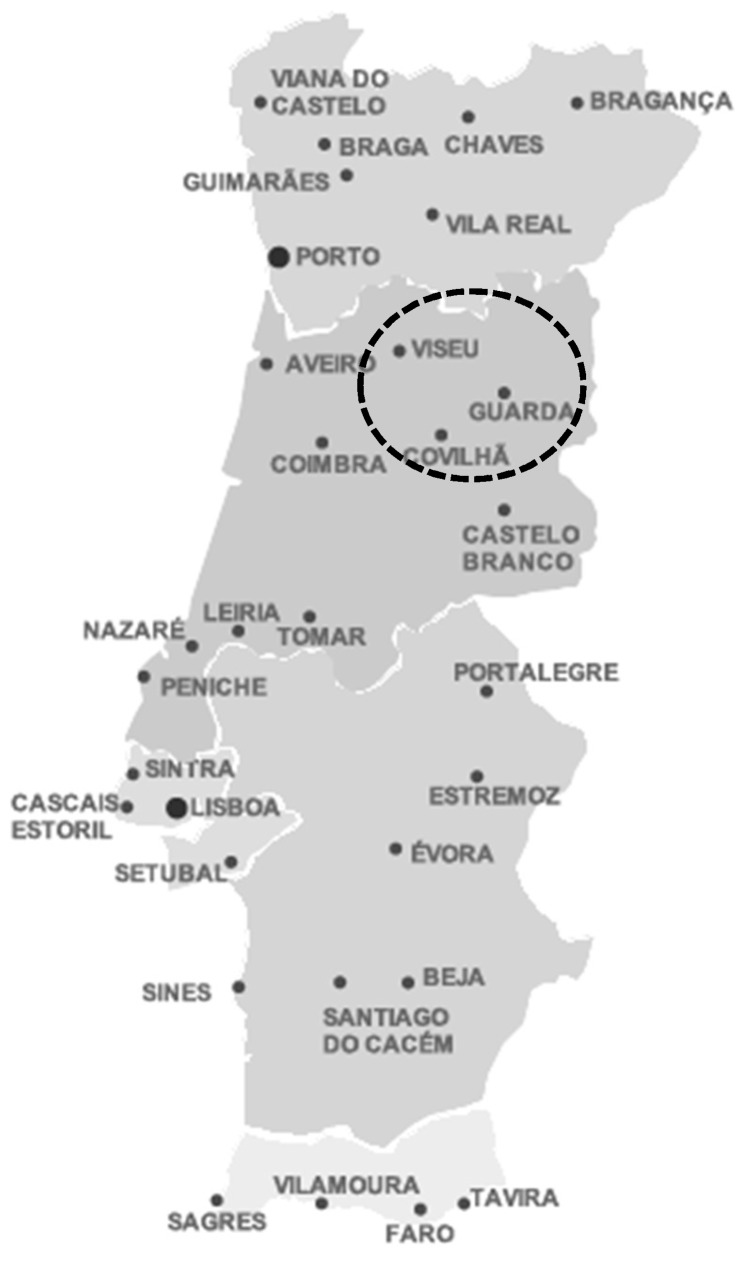
Map of Portugal showing the location of Viseu and Covilhã.

In order to contribute to the deepening the knowledge on this subject, this article presents the results of measurements of indoor pollutants (carbon dioxide (CO_2_), formaldehyde (CH_2_O), and total volatile organic compounds (TVOC)), as well as hygrothermal comfort conditions (T_int_, RH, and ACH) in three ECC in the cities of Viseu and Covilhã, Portugal; see Figure 1 [15,16].

## 2. Physical Characterization of the Studied Buildings and Compartments

### 2.1. City of Viseu

Portuguese thermal regulations [17] indicate approximately 1700 heating degree days (base 18 °C) for the town of Viseu, with an average elevation of 480 m.

Two ECC with the following conditions were analyzed: (1) ECC 1: 7 rooms and 1 living room; (2) ECC 2: 5 rooms and 3 living room.

A physical characterization of the two buildings and some of the compartments studied is presented in Table 1, which shows that they were constructed at quite different times and in different locations, and feature dissimilar characteristics and technical installations.

According to the regulations in Portugal [18], the minimum area of living rooms should respect a ratio of 2 m^2^/resident. In the case of ECC 2, this ratio is grossly disregarded.

Information about the interior cladding of the studied compartments, windows, and corresponding indoor and outdoor solar protection is presented in Table 2.

### 2.2. City of Covilhã

Portuguese thermal regulations [17] indicate approximately 2000 heating degree days (base 18 °C) for the town of Covilhã, with an average elevation of 750 m.

A physical characterization of the studied building (ECC 3) and compartments is presented in Table 3.

Information about the interior cladding of the studied compartments, windows, and corresponding indoor and outdoor solar protection is presented in Table 4.

## 3. Regulatory Requirements, Standards, and Experimental Conditions

The protective thresholds for the considered physico-chemical pollutants are set out in Table 5.

## 4. Materials and Methods

### 4.1. Experimental Conditions in the City of Viseu

Measurements of exterior temperature were obtained from [25]. Regarding the location chosen for placing the equipment inside the compartments, places were avoided, whenever possible, that could influence the measurements of the indoor environmental parameters, such as windows and climate control equipment. The ACH was assessed using the decay technique, using metabolic CO_2_ as a tracer gas (in post-occupancy periods), as described in ASTM E741 [26] and ASTM D6245 [27]. In Portugal, one of the supporting documents for testing is Technical Note TN-SCE-02, 2009 (ADENE—Portuguese energy agency), which, in Annex 3, allows the use of “Photonionization Detectors” (PID) as a monitoring method for TVOC and formaldehyde measurement [24]. Thus, such equipment was used in both cities.

The measurements of pollutants were carried out continuously in the rooms. They were performed during the period of occupancy [7:30 a.m. to 7:30 p.m.] in the living rooms. In view of the available equipment, only one point of analysis of the various parameters in each compartment was considered [24].

Two trials were conducted: the first in winter, i.e., between December 2015 and January 2016, and the second in spring, i.e., between March and April 2016.

Table 6 presents the parameters, measuring instruments, and the main conditions of measurement.

### 4.2. Experimental Conditions in the City of Covilhã

Outdoor temperature measurements were obtained from a meteorological station at the University of Beira Interior (UBI), located at approximately 680 m altitude. Regarding the location chosen to place instruments inside the compartments and to assess the ACH, the criteria already presented were followed.

The measurements of the pollutants were performed continuously; each reading was measured over a minimum period of 5 min [24]. Due to the limitations of the instruments, the measurements of formaldehyde were punctual, although a maximum of 3 measurements, taken at 5 min intervals, was obtained. The first trial took place in February and June 2014, and the second in April and May 2015.

The minimum number of analysis points for the various indoor air quality parameters to be measured was calculated by applying the following expression to the total area of the compartment, rounding up to the unit [24]:

(1) $$N_{i}\;=\;0.15\;\times \;A_{i}^{0.5}$$

where *N*_i_ is the number of measurement points in zone *i* and *A*_i_ is the area of zone *i* (m^2^).

Table 7 shows the minimum number of measurement points calculated.

Table 8 shows the parameters, measuring instruments, and the main conditions of measurement.

In all presented results (cities of Viseu and Covilhã), the calculated mean (*µ*) refers to the arithmetic mean.

## 5. Presentation and Critical Analysis of Results

In the analysis of the results, according to Table 5, the following situations of discomfort and levels of pollutants were considered inadequate and excessive (the ECC analyzed were considered to be existing buildings [19]): (1) Percentage of time analyzed greater than or equal to 20% in the case of *T*_int_ outside the range [20 °C; 25 °C] and *RH*_int_ outside the range [30%; 70%]; (2) Threshold of CO_2_ protection exceeded (1625 ppm for a mean of 8 h); (3) Threshold of TVOC protection exceeded (0.52 ppm for a mean of 8 h); (4) Threshold of CH_2_O protection exceeded (0.08 ppm for a mean of 8 h).

As for *T*_int_ and *RH*_int_, this “excessive” percentage is considered to be reasonable in assessments of indoor environments, since existing regulations are relatively recent, and the buildings have envelope and HVAC systems that are mostly unprepared for the new, very high standards. Moreover, if the percentage defined is too small (e.g., <5–10%), implementing climate control in these spaces would become quite expensive.

### 5.1. Viseu

#### 5.1.1. ECC 1

In the compartments of ECC 1, in both trials, approximately constant temperatures and relative humidity were recorded; they were within the recommended values (20 °C–25 °C; 30–70%). This may be because the building in question is recent, with improved systems relative to the ECC 2. An example of this is the existence of double-glazed windows with aluminum frames, without thermal brakes, in all of the compartments, as well as the existence of thermal insulation in the envelope of the building.

Table 9 shows a statistical analysis of indoor pollutants (CO_2_ and TVOC), recorded in the compartments of ECC 1 during the second trial.

**Figure 2:**
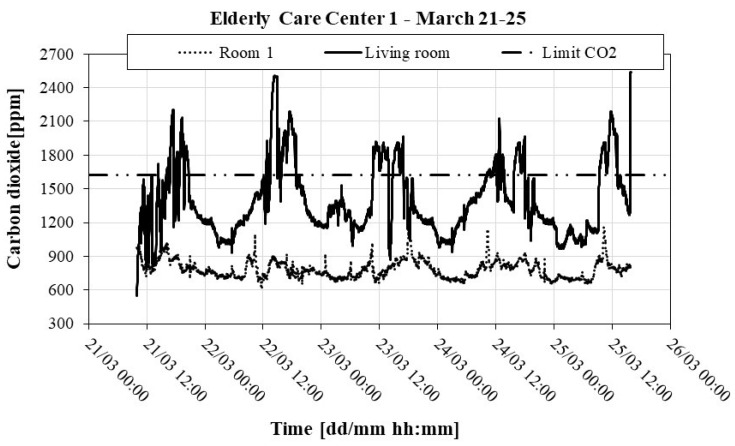
Recording of CO_2_ in room 1 and the living room of ECC 1: 2^nd^ trial (21 to 25 March).

According to the results presented in Table 9 and shown in Figure 2, we conclude that: (1) The living room presented a maximum mean value of CO_2_ which is above the protection threshold. From Figure 2, we can see that the maximum peaks of pollutants occurred during the period of the room’s occupancy, mostly during the afternoon. We can also see that outside the period of occupancy, the values of CO_2_ remained below the regulatory limits. The high values may therefore be due to the high density of occupancy of the living room, reduced ACH, or even to possible combustion processes, since the kitchen and the dining hall are located in the vicinity of the living room; (2) TVOC concentrations were not of concern in any of the analyzed compartments. The standard deviation values of the TVOC of the rooms were high, and we may conclude that the most influential parameters (type of activities and ACH) vary quite a bit during occupancy.

#### 5.1.2. ECC 2—1^st^ Trial

Table 10 shows a statistical analysis of the temperatures and relative humidity recorded in the compartments of ECC 2 during the first trial. We may essentially conclude that: (1) The rooms analyzed show an excessive percentage of time in which the indoor temperatures are lower than prescribed (recommended range: 20 °C to 25 °C). However, comparing these with the mean temperatures, we can state that temperatures which are uncomfortably low are only slightly lower than 20 °C; (2) The temperatures in the living rooms were found to be within the recommended guidelines. These conditions of thermal comfort may reflect better climate control and the fact that these compartments had a high occupancy density throughout the day; (3) The living rooms had reasonable mean *RH* values; (4) The rooms had excessive percentages of time where the *RH* values registered were higher than the recommended maximum limit.

**Figure 3:**
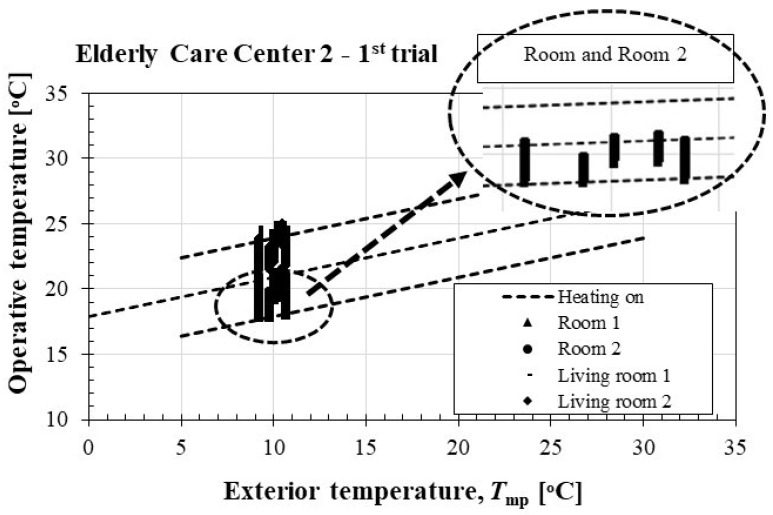
Assessment of thermal comfort by the adaptive method of the LNEC in ECC 2: 1^st^ trial.

The thermal comfort of rooms 1 and 2 and living rooms 1 and 2 of ECC 2 was assessed using the adaptive model proposed by LNEC [28], as shown in Figure 3.

The “Operative temperature” can be calculated, with good approximation, considering the arithmetic mean between *T*_int_ and *T*_mp_ (ASHRAE 55, 2004 [29]; EN ISO 7730, 2005 [30]).

Considering the use of active air conditioning (central heating with water radiators), the results revealed the existence of a thermally comfortable environment in practically all compartments, with the temperature data within the established “comfortable” guideline values.

The statistical analysis of the indoor pollutants (CO_2_ and TVOC) recorded in the compartments of ECC 2 during the first trial showed that: (1) Room 1 and living room 2 had high mean CO_2_ values. Nevertheless, none of the compartments analyzed presented mean TVOC values of concern; (2) With the exception of living room 1, all the compartments analyzed had a maximum mean value of CO_2_ which was above the protection threshold.

Table 11 presents the ACH of living rooms 1 and 2 in ECC 2.

From the results presented, we may conclude that both living rooms analyzed had poor ACH (well below the regulatory ACH in both cases).

### 5.2. Covilhã

#### 5.2.1. 1^st^ Trial

Table 12 shows the results of the pollutant measurements.

With respect to the values presented, and taking the measurement period into account (≈ 5 min), we may conclude that the triple room presented elevated levels of formaldehyde. No potential source was identified; therefore, we speculate that this may be due to the wall coverings or other materials used. These values require further investigation.

**Figure 4:**
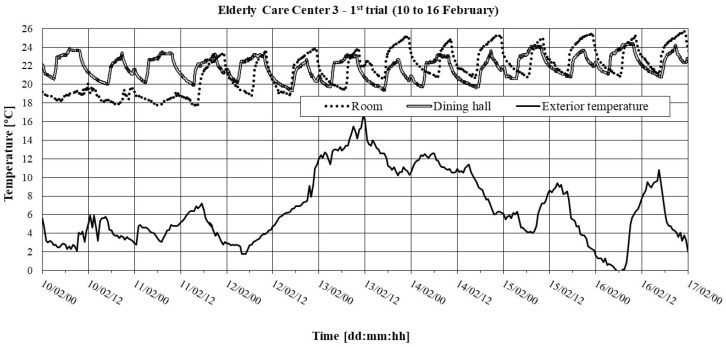
Interior temperature in ECC 3: 1^st^ trial (10 to 16 February).

The hygrothermal comfort conditions are shown in Figure 4 and Table 13.

It should be noted that in the ECC 3, the measurement in the living room was not performed simultaneously with the other two compartments, which is why this compartment is not shown in Figure 4.

An analysis of Figure 4 and Table 13 leads us to conclude that: (1) A cyclical variation of the interior temperature is noticeable. This variation is due to the heating schedule, i.e., heating periods start at 6.00 a.m. and 6.00 p.m.; (2) The compartments present reasonable mean values for the mean indoor temperature, reflecting, in particular, the existing climate control/heating systems; (3) There is an excessive percentage of time with temperatures below regulations in the room (perhaps reflecting the opening of windows while cleaning); (4) There was some time in the two compartments in which the temperatures were above those prescribed by regulation. This may be due to their orientation (SE and E) or insufficient sun protection with regards to the windows, as in the living room; (5) The compartments do not present levels of RH of concern.

#### 5.2.2. 2^nd^ Trial

The 2^nd^ trial took place in the spring of 2015, continuously recording concentrations of CO_2_ and TVOC only at one point, with a 2-min time interval and a “Maximum measurement period” between 1 and 8 days. Table 14 shows the results of the measurements of pollutants and ACH.

**Figure 5:**
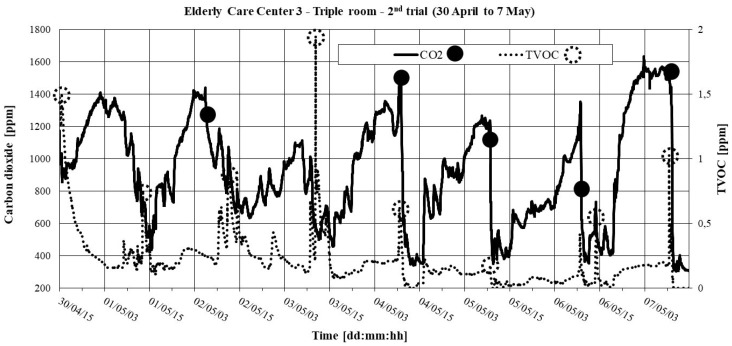
CO_2_ and TVOC in the triple room in ECC 3: 2^nd^ trial (30 April to 7 May).

**Figure 6:**
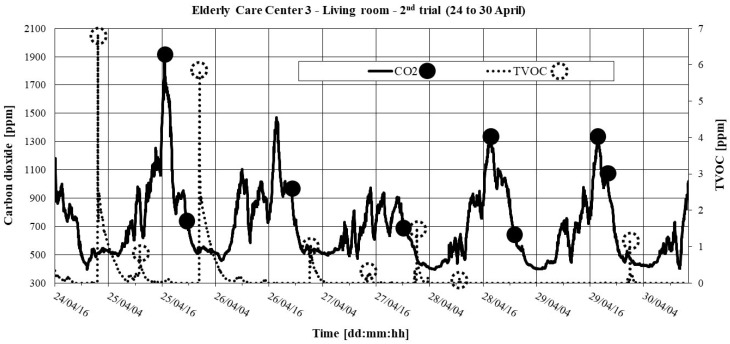
CO_2_ and TVOC in the living room in ECC 3: 2^nd^ trial (24 to 30 April).

Figure 5 and Figure 6 show the temporal evolution of CO_2_ and TVOC. The initial points of the CO_2_ decay method are shown; they were used to determine the ACH through the decay technique. Points with maximum levels of TVOC are also shown.

From Table 14 and Figure 5 and Figure 6, we may conclude that: (1) The living room presents very low values of ACH; (2) In the triple room, the peaks of CO_2_ and TVOC occurred predominantly between 10:00 and 11:00 a.m., followed by a marked decay of CO_2_. This conjugation of behavior may be the result of cleaning actions, followed by opening windows.

## 6. Conclusions and Recommendations

From the results obtained in both ECC in Viseu, the following conclusions may be drawn: (1) With the exception of the concentration of CO_2_ in the living room, ECC 1 has an IEQ which is within the regulatory threshold values and standards; (2) In ECC 2, the rooms have high percentages of time with temperatures below regulation standards, and reveal worrying RH values (with excessive percentages of time with RH values above the recommended maximum limit); (3) In ECC 2, the rooms have excessive periods with temperatures below regulation, and reveal RH values that raise some concerns (with excessive periods of time with RH values above the recommended maximum limit); (4) With the exception of living room 1, all rooms in ECC 2 had maximum mean values of CO_2_ which were above the protection threshold; (5) In assessing the thermal comfort by applying the adaptive comfort model proposed by LNEC and regarding ECC 2, the rooms are comfortable overall; (6) The ACH were found to be insufficient, with values generally below the required levels for all of the analyzed compartments.

From the results obtained in the ECC in Covilhã, the following conclusions stand out: (1) The RH values in the compartments do not raise concern; (2) The triple room has an excessive percentage of time with below regulation temperatures (perhaps due to opening windows during cleaning actions); (3) The living room has very low ACH values; (4) The triple room has high levels of formaldehyde.

The previous conclusions imply the need for action concerning the heating, maintenance, and ventilation conditions, so that the spaces operate within the appropriate conditions of comfort and air quality.

## Figures and Tables

**Table 1:** Viseu: physical characterization of the buildings and some of the studied compartments.

Buildings				Rooms			
	Year of Construction	Climate Control/Heating System	Ventilation System	Designation	Predominant Orientation	Area [m^2^]	Usual Maximum Occupancy/No. of Residents
ECC 1 ^a^	1994	Intermittent heating with water radiators	NV	Room 1	NW	20.58	1
				Room 2	SE	20.58	2
				Room 3	NW	20.58	2
				Living room	SE and SW	257.32	25
ECC 2 ^b^	1869	Intermittent heating with water radiators	NV	Room 1	E and NE	24.61	3
				Room 2	W	22.79	3
				Living room 1	W	39.76	25
				Living room 2 ^c^	W	39.76	25

Notes: ^a^ Cleaning method: daily with aqueous solution; opening of windows during cleaning (unsystematic); ^b^ Cleaning method: daily with aqueous solution; opening of windows during cleaning (unsystematic), outer walls uninsulated; ^c^ Also serves as dining hall; the natural ventilation system (NV) is due to the natural air permeability of the envelope. There is not a properly designed, natural ventilation system.

**Table 2:** Viseu: interior cladding, windows, and solar protection of the various compartments.

Buildings	Compartments	Flooring	Walls	Ceiling	Solar Protection		Windows
					Interior	Exterior	
ECC 1	Room 1	Wooden parquet	Plastered	Plastered	Blackout curtains	Blinds	Aluminum without thermal brake; side hung windows; double glazing
	Room 2						
	Room 3						
	Living room	Ceramic tile			Vertical blinds with fabric slats	None	Aluminum without thermal brake; sliding windows; double glazing
ECC 2	Room 1	Vinyl	Plastered	Plastered	Blackout curtains + semi-opaque curtains	None	Aluminum without thermal brake; side hung windows; double glazing
	Room 2						
	Living room 1						
	Living room 2				Blackout fabric roller blinds		

**Table 3:** Covilhã: physical characterization of the studied compartments.

Building				Compartments			
	Year of Construction	Climate Control/Heating System	Ventilation System	Designation	Predominant Orientation	Area [m^2^]	Usual Maximum Occupancy/No. of Residents
ECC 3	2002	Intermittent heating with water radiators; HVAC in living room and dining hall	RR in the living room and dining hall: MV-intermittent extraction	Living room	E	134.00	25
				Dining hall	E	134.00	50
			RR of the rooms: NV-opening of windows	Triple room	SE	33.92	3

Note: Cleaning method—daily with aqueous solution; opening window while cleaning.

**Table 4:** Covilhã: interior cladding, windows, and solar protection in the various compartments.

					Solar Protection		
Building	Compartments	Flooring	Walls	Ceiling	Interior	Exterior	Windows
ECC 3	Room	Vinyl and ceramic tile	Ceramic tile	Plasterboard	Slightly transparent curtains	Blinds	Aluminum without thermal brake; sliding windows; double glazing
	Living room/dining hall	Vinyl			Semi-opaque fabric roller blinds	None	

**Table 5:** Parameters measured and reference concentrations.

Parameters		Protection Threshold in New Buildings		Margin of Tolerance ^b^ (*MT* ^c^)	Protection Threshold ^b^	
		[mg/m^3^]	[ppmv]	[%]	[mg/m^3^]	[ppmv]
Physico-chemical pollutants	TVOC [19]	0.6	0.26 ^a^	100	1.2	0.52 ^a^
	CH_2_O [19]	0.1	0.08			
	CO_2_ [19]	2250	1250	30	2925	1625
		Recommended/regulated level				
Hygrothermal comfort	*T* [20,21,22]	20–25 °C				
	*RH* [22,23]	30–70%				

Notes: ^a^ Value obtained for the molar mass of isobutylene [24]; ^b^ Margin of tolerance and protection threshold in existing buildings and new buildings without mechanical ventilation systems; for a mean of 8 h; ^c^ MT is the value added to “Protection threshold in new buildings” to get “Protection threshold in existing buildings and new buildings without mechanical ventilation”, e.g., for TVOC: 0.6 mg/m^3^ × 100% = 1.2 mg/m^3^.

**Table 6:** Viseu: parameters, technical characteristics of the equipment used, and conditions of measurement.

Parameters	Instrument	Accuracy	Measurement Range	Duration of Measurement	Maximum Measurement Period ^a^
*T* *RH* CO_2_	Telaire 7001 with data logger U12-013 coupled to record data	±0.35 °C (0 to 50 °C); ±2.5% (10 to 90%); ±50 ppm	−20 to70 °C; 5 to 95%; 0 to 10,000 ppm on the display; 0 to 4000 ppm on the external connection	1 min	4 days
*T* *RH*	Data logger U12-012	±0.35 °C (0 to 50 °C); ±2.5% (10 to 90%)	−20 to 70 °C; 5 to 95%	1 min	4 days
*T* *RH* CO_2_	Fluke 975 AirMeter	±0.9 °C (40 to 60 °C); ±0.5 °C (5 to 40 °C); ±1.1°C (−20 to 5 °C); ±2% (10 to 90%); 2.75% + 75 ppm	−20 to 60 °C; 10 to 90%; 0 to 5000 ppm	1 min	4 days
TVOC	PhoCheck Tiger (PID)	±5% of the display reading ± one digit	1 ppb to 20,000 ppm	30 s	10 h

Note: ^a^: During an experimental campaign, there are several measurement periods (e.g., depending on the equipment’s memory capacity or the experimental conditions); the “Maximum measurement period” is the longest measurement period.

**Table 7:** Minimum number of measurement points per compartment.

Building	Compartment	Area [m^2^]	Volume [m^3^]	Minimum Number of Measuring Points	Number of Measured Points
ECC 3	Living room	134.00	375.2	2	3
	Canteen	134.00	375.2	2	3
	Triple room	33.92	95.0	1	1

**Table 8:** Covilhã: parameters, technical characteristics of the equipment used, and conditions of measurement.

Parameters	Instruments	Accuracy	Measuring Range	Measuring Interval ^a^	Measuring Period ^b^
CH_2_O	Formaldemeter htV-M	±10%	0–10 ppm	Punctual ^a^	3 measurements spaced every 5 min
CO_2_	TSI Velocicalc 9565-P (986 probe)	±3% or 50 ppm, whichever is greater	0–5000 ppm	15 s	3 measurements 5 min each
TVOC	TSI Velocicalc 9565-P (986 probe; PID)	±20% (according to the representative’s information)	0.01–20 ppm, isobutylene	15 s	3 measurements 5 min each
*T* *RH*	Extech RH520	±1.0 °C; ±3.0%	–28–60 °C; 10–95%	1 min	15 to 30 days

Notes: ^a^ “Measuring interval” is the time between two successive measurements. The formaldehyde measuring interval spot (punctual) is an isolated (instantaneous) measurement; ^b^ “Measuring period” is the set of all “Measuring intervals”.

**Table 9:** Statistical analysis of the pollutants recorded in ECC 1 compartments: 2^nd^ trial (21 to 25 March).

	CO_2_ [ppm]			TVOC [ppm]		
Compartment	Max	*μ* ± *σ*	Maximum of 8 h Means	Max	*μ* ± *σ*	Maximum of 8 h Means (*n* = 480)
Room 1	1272	78 ± 83	888	0.86	0.05 ± 0.06	0.05
Room 2	1343	710 ± 101	840	2.60	0.20 ± 0.25	0.24
Room 3	1324	705 ± 245	850	0.32	0.07 ± 0.06	0.08
Living room	2538	1562 ± 335	1861	0.08	0.04 ± 0.02	0.04

Notes: (1) The measurements were continuous in the rooms, while in the living room, the measurement refers to the normal period of occupancy: from 7.30 a.m. to 7.30 p.m.; (2) The mean concentration of CO_2_ and TVOC were calculated without taking into account any errors (e.g., external or equipment); (3) “Maximum of 8 h means” is the maximum value that occurs in sequentially-calculated 8 h means. “Maximum of 8 h means” occurs during “Maximum measurement period” (See Table 6). Approximately 5500 arithmetic means were calculated.

**Table 10:** Statistical analysis of the hygrothermal parameters recorded in compartments of ECC 2: 1^st^ trial (4 to 8 January).

	*T*_int_ [°C]			*RH*_int_ [%]			*T*_ext_ [°C]	Δ*T* [°C]
Compartment	*μ* ± *σ*	Perc ≤ 20 °C	Perc ≥ 25 °C	*μ* ± *σ*	Perc ≤ 30%	Perc ≥ 70%		
Room 1	19.6 ± 0.9	64	0	73 ± 1	0	99	9.9 ± 2.6	9.7
Room 2	19.9 ± 0.8	55	0	70 ± 2	0	51		10.0
Room 3	20.4 ± 0.8	31	0	68 ± 2	0	21		10.5
Room 4	19.5 ± 0.8	74	0	69 ± 3	0	49		9.6
Room 5	19.7 ± 0.7	63	0	69 ± 3	0	46		9.8
Living room 1	24.4 ± 0.3	0	3	52 ± 4	0	0	10.1 ± 2.5	14.3
Living room 2	23.1 ± 0.8	0	0	59 ± 2	0	0		13.0
Living room 3	22.4 ± 0.5	0	0	57 ± 5	0	0		12.3

Notes: (1) The measurements were continuous in the rooms, while in the living room, the measurement refers to the normal period of occupancy: from 7.30 a.m. to 7.30 p.m.; (2) Δ*T* is the difference between the means of *T*_int_ and *T*_ext_; (3) *Perc* is “Percentage of time that exceeds a certain value”; see Nomenclature.

**Table 11:** Ventilation rates of living rooms 1 and 2 in ECC 2: 1^st^ trial.

Compartment	ACH_av_ [h^−1^] ^a^	*q*_RECS_ [m^3^/(h-person)] ^b^	Volume [m^3^]	Maximum Usual Occupancy—Number of Residents	ACH_RECS_ [h^−1^] ^c^
Living room 1	0.48	24	117.69	25	5.10
Living room 2	0.39	24	71.97	20	6.67

Notes: ^a^ To determine mean ACH by the CO_2_ decay method, a total of 5 trials were used for living room 1 and 4 for living room 2; ^b^ Air flow rates were obtained in [19]; *q*_RECS_ means “Air flow rate”, obtained in accordance with “Ordinance 353-A/2013” (see Nomenclature); ^c^ ACH_RECS_ means “Air change rate“, obtained in accordance with “Ordinance 353-A/2013” (see Nomenclature).

**Table 12:** Statistical data of the pollutants recorded in ECC 3 compartments: 1^st^ trial (March).

	Compartment	CO_2_ [ppm]_Max_^b^	TVOC [ppm]_Max_^b^	CH_2_O [ppm]_Max_^b^
ECC 3	Living room	889	0.25	0.07
	Dining hall	694 ^a^	0.19	0.06
	Triple room	684 ^a^	0.33	0.13

Notes: ^a^ measurement obtained outside hours of use; ^b^ maximum value of 3 means of 5 min each.

**Table 13:** Statistical analysis of hygrothermal parameters recorded in compartments of ECC 3: 1^st^ trial (February–March).

		*T*_int_ [°C]			*RH*_int_ [%]			*T*_ext_ [°C]	Δ*T* [°C]
	Compartment	*μ* ± *σ*	Perc ≤ 20 °C	Perc ≥ 25 °C	*μ* ± *σ*	Perc ≤ 30%	Perc ≥ 70%	*μ* ± *σ*	
ECC 3	Living room	23.8 ± 1.2	7	16	44 ± 5	0	0	10.4 ± 4.9	13.4
	Dining hall	22.7 ± 1.3	2	1	46 ± 5	0	0	7.4 ± 3.3	15.3
	Triple room	22.0 ± 2.3	26	11	47 ± 7	1	0	7.4 ± 3.3	14.6

**Table 14:** Statistical analysis of the pollutants recorded in ECC 3 compartments and calculation of ACH: 2^nd^ trial (April–May).

		CO_2_ [ppm]		ACH_av_ [h^−1^]	ACH_RECS_ [h^−1^]	TVOC [ppm]		CH_2_O [ppm]_Max_
	Compartment	*μ* ± *σ*	Perc ≥ 1625			*μ* ± *σ*	Perc ≥ 0.52	μ
ECC 3	Living room	723 ± 253	0.8	0.22	1.6	0.04 ± 0.09	6.7	0.09
	Triple room	890 ± 335	0.0	0.81	0.5	0.17 ± 0.15	7.3	0.04

Notes: (1) The measurements in the living room refer to the usual period of occupancy: 6.30 a.m. to 6.30 p.m.; (2) A total of 8 trials were conducted in the living room, and 5 in the triple room, to determine the mean ACH by the CO_2_ decay method.

## Nomenclature

*A*	Area of a zone, [m^2^]
ACH	Air change rate, [h^−1^]
CH_2_O	Formaldehyde
CO_2_	Carbon dioxide
E, NE, NW, SE, SW, W	Cardinal points: E—East; NE—Northeast; NW—Northwest; SE—Southeast; SW—south-west; W—West
ECC	Elderly Care Centers
HVAC	Heating, Ventilation, and Air Conditioning
IAQ	Indoor Air Quality
IEQ	Indoor Environment Quality
I/O	Indoor/Outdoor ratio
LNEC	National Laboratory for Civil Engineering
Max	Maximum
Min	Minimum
*MT*	Margin of Tolerance
MV	Mechanical Ventilation
N	Number of measurement points
n	Number of values measured to average 8 h
NV	Natural Ventilation
Perc	Percentage of time that exceeds a certain value
*q*	Air flow rate, [m^3^/h]
*RH*	Relative Humidity, [–]
RR	Restroom
*T*	Temperature, [°C]
*T*_mp_	Running mean outdoor temperature, [°C]
TVOC	Total Volatile Organic Compounds
VOC	Volatile Organic Compound
*Greek symbols*	
Δ	Difference
*μ*	Mean
*σ*	Standard deviation
*Subscripts*	
av	Average (arithmetic mean)
ext	Referring to the exterior
i	Referring to a zone
int	Referring to the interior
Max	Maximum
RECS	Referring to Ordinance 353-A/2013
